# Development and Validation of an Item Bank for Depression Screening in the Chinese Population Using Computer Adaptive Testing: A Simulation Study

**DOI:** 10.3389/fpsyg.2018.01225

**Published:** 2018-07-18

**Authors:** Qingrong Tan, Yan Cai, Qiuyun Li, Yong Zhang, Dongbo Tu

**Affiliations:** School of Psychology, Jiangxi Normal University, Nanchang, China

**Keywords:** computerized adaptive testing, item response theory, depression assessment, IRT models, measurement

## Abstract

With the increasing prevalence of depression, creating a simple and precise tool for measuring depression is becoming more important. This study developed a computer adaptive testing for depression (CAT-Depression) from a Chinese sample. The depression item bank was constructed from a sample of 1,135 participants with or without depression using the Graded Response Model (GRM; Samejima, 1969). The final depression item bank with strict unidimensionality comprised 68 items, which had local independence, good item-fit, high discrimination, no differential item functioning (DIF), and each item measured at least one symptom of diagnostic criteria for depression in *ICD-10*. In addition, the mean IRT discrimination of the item bank reached 1.784, which clearly showed that the item bank of CAT-Depression was high-quality. Moreover, a simulation CAT study with real response data was conducted to investigate the characteristics, marginal reliability, criterion-related validity, and predictive utility (sensitivity and specificity) of CAT-Depression. The results revealed that the proposed CAT-Depression had acceptable and reasonable marginal reliability, criterion-related validity, and sensitivity and specificity.

## Introduction

Depression is one of the most prevalent psychological and behavioral disorders, and the number of people who commit suicide because of depression is growing. By the year 2020, depression will account for 5.7% of the total burden of disease (Dennis et al., 2016), and will be the second greatest disease leading to disability and death after coronary heart disease according to the World Health Organization (Dennis and Hodnett, 2014). At present, the number of depressed patients who choose to seek medical treatment is growing, thus, it is very essential to have accurate assessment and diagnosis of patients with depression and provide timely treatment.

In the past, evaluation of depression was predominantly based on questionnaires that were compiled according to the classical test theory (CTT) framework. These questionnaires include the Center for Epidemiological Studies-Depression Scale (CES-D; Radloff, 1977), the Self-rating depression scale (SDS; Zung, 1965), the Patient health questionnaire (PHQ-9; Kroenke et al., 2001), and the Beck Depression Inventory (BDI; Beck et al., 1987). These questionnaires under the CTT framework have fixed lengths, and usually contain items corresponding to various levels of depression. A large number of items may deviate from the symptoms of respondents with depression, in that respondents are commonly required to answer each item of a questionnaire, which may increase patients' unnecessary measurement burden, therefore, reducing respondents' enthusiasm. Moreover, these cannot provide respondents with more information about their depressive state.

In recent decades, a large number of researchers have used item response theory (IRT) to improve existing depression scales. For example, Aggen et al. (2005) used the Rasch model and a 2-parameter logistic (2PL) model to test the psychometric characteristics of major depression in the Diagnostic and Statistical Manual of Mental Disorders (DSM-III-R), and Stansbury et al. (2006) also applied the IRT model in an analysis of the CES-D scale. The latest and probably most fascinating new perspective provided by IRT is computerized adaptive testing (CAT), which is a form of testing that uses a computer to automatically select appropriate items for the examinee (Almond and Mislevy, 1998). In brief, CAT selects an item that is appropriate to the examinee's theta from an item bank, then updates the examinee's theta according to the responses to this item. This process is repeated until the examinee's theta is accurately estimated. We have found that CAT is an effective way to administer items. Several studies have shown that CAT can largely limit items administered, to reduce patients' burden without loss of measurement accuracy and can save patients and diagnostician considerable time. In addition, examinees' motivation to respond increased, because the selected items corresponded highly to the examinee's theta (Gibbons et al., 2008), and the examinee may think that the test was tailored for their own condition. Furthermore, the test administrator could control the standard error (SE) of the measurement and reduce test length with negligible loss of reliability and measurement precision (Gershon, 2005). CAT also has disadvantages, such as being a complex technique, having high initial costs, and requiring a substantial amount of human and financial resources to organize a CAT program. However, the advantages significantly outweigh the disadvantages (Meijer and Nering, 1999).

Regarding CAT for depression, different versions have been researched. For example, Gardner et al. (2004) used the Graded Response Model (GRM; Samejima, 1969) to model the BDI and developed a computer-adaptive version of the BDI. Smits et al. (2011) developed a computer-adaptive version of the CES-D using the GRM. Fliege et al. (2005) developed a CAT for depression whose items were from several depression scales using the Generalized Partial Credit Model (GPCM; Muraki, 1997). Gibbons et al. (2012) developed a CAT for depression using the bifactor model. Forkmann et al. (2013) developed a CAT for depression with good screening performance (Forkmann et al., 2009). Flens et al. (2017) developed a Dutch-Flemish version of CAT for depression based on the patient-reported outcomes measurement information system (PROMIS).

Although there were several existing studies on the development of CAT for depression, there are still some issues that need to be further addressed. First, some versions of CAT for depression (e.g., Gardner et al., 2004; Smits et al., 2011) were developed based on only one depression scale, which meant that there were very few items in the item bank tailored for different respondents/patients. Second, methodologically, there are a large number of IRT models that can fit CAT under the framework of IRT. However, few studies have compared different IRT models in their CAT and selected one optimal model to fit the CAT based on the test-level model-fit check or other methodological considerations. Thirdly, the samples in existing studies of CAT for depression were from different countries. For example, Gardner et al. (2004) used a European-American sample, Fliege et al. (2005) and Forkmann et al. (2013) used a German sample, while Smits et al. (2011) used a Dutch sample. However, no studies have used a Chinese sample to develop CAT for depression. More importantly, according to the investigation of the National Health and Family Commission of the People's Republic of China, the prevalence of depression in China ranged from 1.6 to 4.1% in 2015 (National Health and Family Commission of the People's Republic of China, 2015). In other words, there were about 22.4 million to 57.4 million people suffering from depression in China. It is therefore necessary to develop an efficient and accurate CAT to measure and diagnose depression in China.

In this study, we hope to address the aforementioned issues by developing a new, more efficient and accurate CAT for depression (hereby referred to as CAT-Depression) by using a Chinese sample. The items in the CAT-Depression bank were preliminarily selected from ten widely-used depression scales according to the symptom criteria of depression defined in *ICD-10*. The preliminarily selected items measured at least one symptom criterion of depression defined in *ICD-10*. In addition, five commonly polytomously-scored IRT models, that is, GRM, GPCM, Partial Credit Model (PCM; Masters, 1982), Rating Scale Model (RSM; Andrich, 1978), and Nominal Response Model (NRM; Bock, 1972), were compared based on test-level model-fit checks to choose one optimal model to fit CAT-Depression. Then, several statistical analyses, including a unidimensionality check, local independence check, item-level model-fit check, and discrimination and differential item functioning (DIF) analyses were conducted to create the final item bank of CAT-Depression. Items with local independence, high discrimination, good item-fit, no DIF, and that measured at least one symptom criterion of depression in *ICD-10* were included in the final item bank of CAT-Depression with unidimensionality. Finally, a CAT simulation study was carried out to investigate the marginal reliability, criterion-related validity, and predictive utility (sensitivity and specificity) of CAT-Depression.

## Methods

### Participants

Participants included healthy individuals and patients with depression, aged from 13 to 80 (*M* = 30.19, *SD* = 12.23). The healthy individuals were primarily from some social groups and colleges whereas the patients with depression were recruited from eight psychiatric hospitals or mental health centers in China. A total of 1,135 participants were recruited for the study, including 922 healthy individuals and 213 patients with a doctor's diagnosis. Table 1 contains other detailed demographic information. The patients with depression were recruited with the following exclusion criteria: those with a history of psychosis, schizoaffective disorder, or schizophrenia; those with alcohol or drug dependence in the past 3 months but not excluding patients with mood disorder; and those with organic neuropsychiatric syndromes such as Alzheimer disease, Parkinson disease, etc. There were also exclusion criteria for healthy individuals: those with a history of psychosis, schizoaffective disorder, or schizophrenia; those with any psychiatric diagnosis within the past 12 months; and those who received treatment for psychiatric problems within the past 12 months. Any patients or healthy individuals who met any of the exclusion criteria were not chosen to participate in this study.

**Table 1:** Demographic characteristics (*N* = 1,135).

**Variables**	**Category**	**Frequency**	**Percent (%)**
Gender	Male	414	36.50
	Female	719	63.30
	Missing	2	0.20
Age	Under 25 years	497	43.80
	25 and above	521	45.90
	Missing	117	10.30
Region	Rural	728	64.10
	City	403	35.50
	Missing	4	0.40

The present study was carried out following the recommendations of psychometrics studies on mental health at the Research Center of Mental Health, Jiangxi Normal University. The protocol was approved by the Research Center of Mental Health, Jiangxi Normal University. Informed consent was obtained from all participants in accordance with the Declaration of Helsinki. Parental informed consent was also obtained for participants aged below 16.

### Measures

The CAT-Depression originally consisted of 117 items. Based on the depression symptom criteria in the *ICD-10*, items were carefully selected from 10 Chinese-versions of self-rating questionnaires, including the Center for Epidemiological Studies-Depression Scale (CES-D; Radloff, 1977), the Self-rating depression scale (SDS; Zung, 1965), the Patient health questionnaire (PHQ-9; Kroenke et al., 2001), the Beck Depression Inventory (BDI; Beck et al., 1987), the Automatic Thoughts Questionnaire (ATQ; Hollon and Kendall, 1980), the Hospital Anxiety and Depression Scale (HADS; Zigmond et al., 1983), the Minnesota Multiphasic Personality inventory (MMPI; Hathaway and McKinley, 1942), the self-report symptom inventory Symptom checklist 90 (SCL-90; Derogatis, 1994), the Carroll's depression scale (CRS; Hamilton, 1967), and the Brief depression scale (BDS; Koenig et al., 1992). Eighteen items measured behavior-related depressive symptoms in the *ICD-10*, 43 items measured cognition-related depressive symptoms, 34 items measured mood-related depressive symptoms, fourteen items measured somatic-related depressive symptoms, and eight items measured the symptom of suicidal thoughts.

Items that measured at least one symptom criterion of depression defined in the *ICD-10* were preliminarily chosen, and all items employed a 2-week recall period and four response categories. For example, item 13 (*Little interest or pleasure in doing things*) measured the *ICD-10* depression symptom of loss of interest or pleasure; item 51 (*Feeling tired or having little energy*) measured the symptom of lack of energy or excessive fatigue; item 64 (*Poor appetite or overeating*) measured the symptom of appetite change; item 69 (*I'm no good*) measured the symptom of inferiority and loss of self-confidence; item 89 (*Have you thought about ending it all*) measured the symptom of suicidal thoughts.

### Data analysis

Data analysis included two parts: construction of the CAT-Depression item bank, and the CAT-Depression simulation study. The first part was the development of the CAT-Depression item bank, while the second part focused on determining the reliability, validity, and predictive utility (sensitivity and specificity) of CAT-Depression. In the first part of construction of the CAT-Depression item bank, statistical analyses based on IRT were sequentially carried out, including the IRT analyses of unidimensionality, local independence, item fit, discrimination, and DIF.

#### Construction of the CAT-depression item bank

##### Unidimensionality

Unidimensionality is a crucial assumption in IRT, and item banks were regarded as unidimensional if the person's latent trait level of the item measures, rather than other factors, resulted in the person's response. Many IRT models assume unidimensionality, such as the two-parameter Logistic model (2PL) and three-parameter Logistic model (3PL) for dichotomous response data, and the GRM, the GPCM, the PCM, the RSM, and the NRM for polytomous response data. Therefore, it is essential to assess whether the item bank is sufficiently unidimensional (Reeve et al., 2007). Confirmatory factor analysis (CFA) was used to evaluate the unidimensionality of the item bank, and a one-factor model CFA was conducted by using the program Mplus 7.0 (Muthén and Muthén, 2012). In CFA, given that the items were polytomous, we used weighted least squares means and a variance adjusted (WLSMA) estimation method, which has a more precise estimation when the variables are categorical data (Beauducel and Herzberg, 2006; Resnik et al., 2012). If the comparative fit index (CFI) ≥ 0.90, the Tucker-Lewis index (TLI) ≥ 0.90, and the root mean square error of approximation (RMSEA) ≤ 0.08, unidimensionality of the item bank was considered sufficient (Kline, 2010). Items with factor loadings greater than 0.3 and significant at *p* = 0.05 were retained in the development of the item bank in this procedure.

##### Local independence

Local independence is also a vital assumption in IRT. We used Yen's Q3 statistic (Yen, 1993) to evaluate this assumption, and Q3 values higher than 0.36 were considered locally dependent (Flens et al., 2017). Therefore, one item with Q3 > 0.36 in each item pair was deleted in this study. Local independence analysis was conducted via the R package mirt (Version 1.24; Chalmers, 2012).

##### Test fit and IRT model selection

In this study, five polytomously-scored IRT models (i.e., the GRM, the GPCM, the PCM, the RSM, and the NRM) were simultaneously applied to fit the selected items of CAT-Depression, and the optimal model was selected based on test-level model-fit indices for further analysis. The widely-used test-level model-fit indices include−2Log-Likelihood (-2LL; Spiegelhalter et al., 1998), Akaike's information criterion (AIC; Akaike, 1974), and the Bayesian information criterion (BIC; Schwarz, 1978). Smaller values of these test-fit indices indicate better model-fit, thus we selected the model with smaller test-fit indices for the later analysis. Model selection was conducted by using the software flexMIRT (Version 3.51; Cai, 2017).

##### Item fit

Item fit was evaluated by the S-χ^2^ statistic (Kang and Chen, 2008), which quantifies and compares the differences between observed frequencies and expected frequencies under the IRT model. Items with *p* values of S-χ^2^ less than 0.001 were deemed to have poor item-fit (Flens et al., 2017) and were eliminated. In this study, a stricter rule was used instead of the recommendation of Flens et al. (2017): items with *p* values of S-χ^2^ less than 0.01 were deemed to have poor item-fit and eliminated. Item fit was also conducted by using the R package mirt (Version 1.24; Chalmers, 2012).

##### Discrimination

Item discrimination shows the extent to which individuals with similar scores can be differentiated via an item. An item with high discrimination implies that this item is preferable to distinguish whether individuals exhibit signs of depression. Therefore, a high discrimination parameter of one item suggests that this item is of high-quality and is helpful to obtain more precise estimation of a population latent trait. Moreover, item discrimination has an important impact on item information or standard error of measurement, which was used to decide which item was selected in the CAT environment. We used the software flexMIRT (Version 3.51; Cai, 2017) to estimate item parameters via the optimal model based on a test-level model-fit check and chose items with discrimination more than 0.8.

##### Differential item functioning

Given the importance of a questionnaire having no measurement bias in practice, DIF analysis was used here to assess systematic errors due to group bias (Zumbo, 1999). Ordinal logistic regression (Crane et al., 2006) was employed to perform DIF analysis under the optimal model based on a test-level model-fit check via the package lordif (Version 0.3-3; Choi, 2015). Change in McFadden's pseudo *R*^2^ was used to evaluate effect size, and the hypothesis of no DIF was rejected when *R*^2^ change was equal to or greater than 0.2 (Flens et al., 2017). Therefore, these items with changes in McFadden's pseudo *R*^2^ ≥ 0.2 were removed from the final analysis. We evaluated DIF for region (rural, city), gender (male, female) and age (under 25 years, 25 and above) groups.

The IRT analyses of unidimensionality, local independence, item fit, discrimination, and DIF were sequentially performed until all remaining items of CAT-Depression sufficiently satisfied the above rules (i.e., unidimensionality, local independence, good item-fit, high discrimination, and having no DIF). Items that satisfied all the following criteria was included in the item bank of CAT-Depression: (1) measuring at least one depression symptom, (2) satisfying the hypothesis of measuring one main dimension in IRT, (3) satisfying the hypothesis of local independence in IRT, (4) fitting the IRT model well, (5) having high discrimination with more than 0.8, and (6) having no DIF. Subsequently, by using the optimal model based on test-level model-fit check, the item parameter and theta parameter of the final item bank were re-estimated for the further CAT via the software flexMIRT (Version 3.51; Cai, 2017).

#### CAT-depression simulation study

The CAT simulation study with the real participants' responses data in paper and pencil (P&P) was conducted to investigate the characteristics, marginal reliability, criterion-related validity, and predictive utility (sensitivity and specificity) of the CAT-Depression.

##### Starting point

In the CAT simulation, item selection is dependent on the examinee's responses to a given item. However, the examinee knows nothing about prior information at the beginning (Kreitzberg and Jones, 1980). The first item was randomly selected from the final depression item bank (Magis and Barrada, 2017), as this method is simple and effective.

##### Scoring algorithm

After the execution of each item, the examinees' depression theta was updated with the expected a posteriori method (EAP; Bock and Mislevy, 1982) based on his or her real response of the selected item in P&P,

$$\begin{array}{l} \overset{\wedge }{\theta_{i}}=\frac{\sum_{k=1}^{q}\theta_{k}L_{i}\left(\theta_{k}\right)\;\cdot \;A\left(\theta_{k}\right)}{\sum_{k=1}^{q}L_{i}\left(\theta_{k}\right)\;\cdot \;A\left(\theta_{k}\right)}, \end{array}$$

θ_*k*_ refers to the quadrature points and serves as a replacement of the specific ability value. Given an ability value θ_*k*_, *L*_*i*_(θ_*k*_) is the likelihood function of examinee *i* with a specific response pattern, where *A*(θ_*k*_) is the weight of the quadrature points, and $\sum_{k=1}^{q}A\left(\theta_{k}\right)=1$. The calculations of EAP are not complex, are noniterative (Bock and Mislevy, 1982), EAP algorithms are a better choice because of their efficiency and stability.

##### Item selection algorithm

The new item with the highest information at that estimated theta point was selected using the maximum Fisher information (MFI) criterion (Baker, 1992) when the examinee's theta was updated. The Fisher information is then defined as

$$I_{j}\left(\overset{\wedge }{\theta }\right)=\;\sum_{k=1}^{K}\frac{{\left[P_{k^{\prime }}(\overset{\wedge }{\theta })\right]}^{2}}{P_{k}(\overset{\wedge }{\theta })},$$

where $I_{j}\left(\overset{\wedge }{\theta }\right)$ is the item information function of item *j* given the $\overset{\wedge }{\theta }$, where $\overset{\wedge }{\theta }$ is the estimated theta, $P_{k}(\overset{\wedge }{\theta })$ is the probability of getting score k given $\overset{\wedge }{\theta }$, *K* is the total score of item *j*, and $P_{k^{\prime }}(\overset{\wedge }{\theta })$ is the first derivative of $P_{k}\left(\overset{\wedge }{\theta }\right)$ to $\overset{\wedge }{\theta }$. MFI criterion can not only ensure efficiency, but can also actively control measurement error, and is a widely used item selection algorithm.

##### Stopping rule

In this study, the termination rules were based on the standard error (SE) of measurement, which meant the test was terminated if the pre-specified value of SE was met or the item bank was exhausted. SE for a trait level can be defined as the reciprocal of the square root of the value of the test information function at that trait level (Magis and Raiche, 2012),

$$\begin{array}{l} SE\left(\theta \right)=\frac{1}{\sqrt{\sum_{j=1}^{n}I_{j}\left(\theta \right)}}, \end{array}$$

where *n* is the number of items the examinee has answered; the stopping rule ensures the precision of parameter estimation, and makes the test result fair for each examinee. Several cut-off values of SE (theta) were used in the CAT-Depression simulation: all items in the bank administered (None), SE (theta) ≤ 0.2, SE (theta) ≤ 0.3, SE (theta) ≤ 0.4, SE (theta) ≤ 0.5, and SE (theta) ≤ 0.6, respectively. The R (Version 3.4.1; Coreteam, 2015) and R package catR (Version 3.12; Magis and Barrada, 2017) were used here for the above analysis.

##### Characteristics of CAT-depression

To explore the characteristics of the CAT-Depression, several statistics were calculated: the mean and standard deviation (SD) of items used, the mean SE of theta estimates, the Pearson's correlation between the estimated theta in CAT-Depression, and theta estimations using the entire item bank, and the marginal reliability that was the mean reliability for all levels of theta (Smits et al., 2011). In a CAT framework, each individual's information can be obtained based on the administered item parameters and his or her responses to these items. The corresponding reliability of each individual can be derived via the following formula (Samejima, 1994) when the mean and SD of theta are fixed to 0 and 1, respectively,

$$\begin{array}{l} r_{xx}\left(\theta_{i}\right)=1-\frac{1}{I\left(\theta_{i}\right)}, \end{array}$$

the *I*(θ_*i*_) is the test information for the *i*-th individual, while the *r*_*xx*_(θ_*i*_) is the corresponding reliability in IRT for the *i*-th individual. Then, we can calculate the mean reliability of all individuals to get the marginal reliability. Furthermore, we plotted the number of selected items as a function of the final theta estimation and the test information curve under several stopping rules. The test information displays the measurement precision of CAT-Depression, and the greater the value, the smaller the error of the theta estimation.

##### Criterion-related validity and predictive utility (sensitivity and specificity) of CAT-depression

To further investigate the criterion-related validity and predictive utility (sensitivity and specificity) of CAT-Depression, the CES-D, SDS, and PHQ-9 scales, which are widely-used and well-validated in diagnosing depression, were selected as criterion scales. The Pearson's correlations between the estimated theta in the CAT-Depression and the standard scores of the SDS, the sum score of the CES-D, and the PHQ-9 were calculated to address the criterion-related validity of CAT-Depression. Then, the area under (AUC) the receiver operating characteristic curve (ROC) was used as an additional criterion to investigate the predictive utility (sensitivity and specificity) (Smits et al., 2011) of CAT-Depression. We used the CES-D, SDS, and PHQ-9, respectively, as the classified variable for depression, and the estimated theta in CAT-Depression was used as a continuous variable to plot the ROC curve under each stopping rule via the software SPSS 17.0. The AUC is a statistic used to evaluate ROC curve, and its value ranged from 0.5 to 1. A larger AUC value indicates a better diagnostic effect (Kraemer and Kupfer, 2006). The predictive utility of the estimated theta for diagnosing depression is similar to random guessing when AUC = 0.5, while it is perfect when AUC = 1. The AUC ranged from 0.5 to 0.7, from 0.7 to 0.9, and from 0.9 to 1, which indicates the predictive utilities were small, moderate, and high, respectively (Forkmann et al., 2013). Determination of the critical value was calculated by maximizing the Youden-Index (YI = sensitivity + specificity − 1) (Schisterman et al., 2005). Here sensitivity refers to the probability that a patient is accurately diagnosed with a disease, and specificity refers to the probability that general people are diagnosed with no illness; the larger the value of these two indicators, the better the effect of the diagnosis.

## Results

### Construction of the CAT-depression item bank

#### Unidimensionality and local independence

In the one-factor model CFA run in the initial CAT-Depression item bank of 117 items, 23 items were eliminated because the factor loadings were less than 0.3 or not significant at *p* = 0.05. After excluding the 23 items with low factor loadings or non-significance from the item bank, we re-ran the one-factor model CFA based on the remaining 94 items. Results of the one-factor model CFA of the 94 remaining items in the item bank showed acceptable model fit: CFI = 0.902, TLI = 0.900 and RMSEA = 0.051. The results clearly showed that the remaining item bank (including 94 items, see Table 2) met the assumption of unidimensionality. Table 2 shows that 15 items were deemed to be locally dependent as their Yen's Q3 statistic was greater than 0.36, items with local dependence were removed from the item bank.

**Table 2:** Factor loading of the CFA1 and CFA2 and reasons for exclusion.

**Item**	**Assessed symptom**	**Factor loading**	**Factor loading**	**Excluded due to…**	**Item**	**Assessed symptom**	**Factor loading**	**Factor loading**	**Excluded due to…**
		**CFA1**	**CFA2**				**CFA1**	**CFA2**	
1	Mood	0.71	0.71		60	Cognition	0.54		CFA
2	Mood	0.73	0.75	Q3	61	Cognition	0.81	0.82	
3	Mood	0.51	0.52		62	Cognition	0.33	0.37	S-X2+ Discrimination
4	Mood	0.58	0.59	Q3	63	Mood	0.77		CFA
5	Mood	0.75	0.76	Q3+S-X2	64	Somatic	0.47	0.50	
6	Mood	0.59	0.52	Q3	65	Behavior	0.47		CFA
7	Mood	0.67	0.59		66	Cognition	0.61	0.63	
8	Cognition	0.57	0.59		67	Cognition	0.58	0.59	Q3
9	Somatic	0.43	0.44		68	Cognition	0.73	0.74	
10	Somatic	0.22		CFA	69	Cognition	0.78	0.79	
11	Cognition	0.21		CFA	70	Behavior	0.60	0.61	
12	Mood	0.76		CFA	71	Behavior	0.76	0.77	
13	Mood	0.67	0.69		72	Cognition	0.77	0.78	
14	Cognition	0.66	0.67		73	Cognition	0.75	0.76	
15	Mood	0.75	0.76		74	Cognition	0.79	0.79	
16	Mood	0.60	0.62		75	Cognition	0.72	0.73	
17	Mood	0.78		CFA	76	Cognition	0.67		CFA
18	Cognition	0.66	0.61		77	Mood	0.76	0.78	
19	Mood	0.64	0.66	Q3+DIF	78	Cognition	0.67	0.69	
20	Mood	0.6	0.62		79	Cognition	0.71		CFA
21	Mood	0.43	0.45		80	Cognition	0.76	0.77	
22	Mood	0.80	0.8		81	Cognition	0.64	0.65	
23	Cognition	0.63	0.65		82	Mood	0.63	0.64	
24	Mood	0.72	0.74	Q3	83	Cognition	0.83	0.83	S-X2
25	Somatic	0.32		CFA	84	Cognition	0.82	0.82	
26	Mood	0.71	0.72	S-X2	85	Cognition	0.41		CFA
27	Mood	0.48	0.48	DIF	86	Cognition	0.82	0.82	
28	Mood	0.67	0.59		87	Cognition	−0.40		CFA
29	Mood	0.80	0.81		88	Suicide	0.84	0.84	Q3
30	Mood	0.43	0.38	Discrimination	89	Suicide	0.78	0.79	
31	Mood	0.68		CFA	90	Cognition	0.61	0.63	
32	Mood	0.39	0.32	Q3+Discrimination	91	Suicide	0.85	0.84	Q3
33	Behavior	0.62	0.63		92	Suicide	0.87	0.87	Q3
34	Behavior	0.74	0.76		93	Mood	0.49	0.51	DIF
35	Somatic	0.24		CFA	94	Cognition	0.61		CFA
36	Behavior	0.36	0.31	Discrimination	95	Suicide	0.78		CFA
37	Somatic	0.53	0.55		96	Somatic	0.68		CFA
38	Somatic	0.65	0.56	Q3	97	Somatic	0.41	0.41	Q3+ Discrimination
39	Behavior	0.69	0.69		98	Somatic	0.48		CFA
40	Cognition	0.74	0.75		99	Mood	0.80	0.80	
41	Cognition	0.67	0.61		100	Somatic	0.48	0.49	
42	Somatic	0.34	0.36	Discrimination	101	Somatic	0.12		CFA
43	Behavior	0.68	0.70		102	Cognition	0.76	0.78	Q3
44	Behavior	0.60	0.62		103	Behavior	0.59	0.61	
45	Behavior	0.57		CFA	104	Behavior	0.79	0.80	
46	Cognition	0.82	0.82		105	Cognition	0.73	0.75	
47	Somatic	0.50	0.52		106	Suicide	0.78	0.78	
48	Cognition	0.43	0.39	Discrimination	107	Mood	0.72	0.73	
49	Cognition	0.57	0.60		108	Behavior	0.29	0.32	Discrimination
50	Cognition	0.55	0.56		109	Suicide	0.85	0.86	
51	Behavior	0.65	0.67		110	Cognition	0.88	0.88	
52	Mood	0.71	0.72		111	Behavior	0.82	0.83	
53	Behavior	0.72	0.74	Q3	112	Mood	0.52		CFA
54	Cognition	0.59	0.61		113	Cognition	0.65	0.67	
55	Cognition	0.66	0.68		114	Behavior	0.71	0.72	
56	Cognition	0.72	0.73		115	Behavior	0.78	0.79	
57	Mood	0.71		CFA	116	Mood	0.74		CFA
58	Cognition	0.59	0.61		117	Suicide	0.69	0.68	S-X2
59	Cognition	0.60	0.54						

*CFA1, the first CFA run of 117 items; CFA2, the second CFA run of 94 items*.

#### Test fit and IRT model selection

Test fit statistics of the GRM, the GPCM, the PCM, the RSM, and the NRM were documented in Table 3. For the GRM, −2LL = 179,190.50, AIC = 179,942.50, and BIC = 181,835.43. All relative fit indices of the GRM were less than those of the other four IRT models, which suggested that the GRM fitted the data better than others. Therefore, the GRM was finally applied to the IRT analysis and CAT-Depression.

**Table 3:** Test-level model-fit for five polytomously-scored IRT models.

**Model**	**-2LL**	**AIC**	**BIC**
Graded Response Model	179,190.50	179,942.50	181,835.43
Generalized Partial Credit Model	180,630.54	181,382.54	183,275.47
Partial Credit Model	185,706.78	186,272.78	187,697.52
Rating Scale Model	190,184.94	190,378.94	190,867.28
Nominal Response Model	179,792.25	180,920.25	183,759.64

*−2LL,−2Log-Likelihood; AIC, Akaike's information criterion; BIC, Bayesian information criterion*.

#### Discrimination, item model-fit, and differential item functioning

Results of item fit and discrimination indicated that five items did not fit the GRM and the discriminations of eight items were less than 0.8 (see Table 2). Regarding DIF, there was no DIF item in the area group, while there were two DIF items in the gender group, and one DIF item in the age group. For gender level, the values of *R*^2^ change were 0.03 and 0.04 for item 19 and item 93, respectively. For age level, the value of *R*^2^ change was 0.02 for item 27. All in all, 11 items with low discrimination (less than 0.8), did not fit the GRM, or having DIF were removed (see Table 2) from further IRT analysis.

Up until this point, the final item bank for CAT-Depression comprised 68 items after 49 items were excluded for the above psychometric reasons. Table 2 displays the eliminated items and reasons for elimination.

Table 4 displays the item parameters of the final item bank of CAT-Depression. The discrimination parameter of the final item bank ranged from 0.84 to 3.14 with an average value of 1.784, which clearly showed that the final item bank of CAT-Depression was of high quality.

**Table 4:** Item parameters of the final item bank with GRM.

**Item**	**Abbreviated item content**	**A**	**b1**	**b2**	**b3**
1	Pessimism	1.68	−0.67	1.40	2.49
2	Happy as others	1.03	−1.13	0.84	2.27
3	Happiness	1.24	−1.57	0.47	2.38
4	Being reproached	1.30	−0.15	2.24	4.12
5	Loss of appetite	0.84	0.13	3.17	5.39
6	Loss of interest	1.62	−0.99	1.97	2.97
7	Being despised	1.69	−0.06	1.91	3.27
8	Still depressed with others' help	2.05	0.02	1.45	2.45
9	Agitation	1.39	−1.17	1.48	3.16
10	Good as others	1.29	−1.13	0.66	2.34
11	Boring	1.40	−1.16	1.00	2.67
12	Social withdrawal	0.88	−0.37	2.06	3.63
13	Loss of pleasure	2.32	0.36	1.62	2.50
14	Concentration difficulty	1.51	−0.74	1.30	2.73
15	Interested in everything around	1.27	−1.76	0.02	1.99
16	Gloomy mood	2.49	−0.57	1.17	2.24
17	So tired and unable to do anything	1.47	−0.51	1.75	3
18	Everything is laborious	2.04	−0.31	1.39	2.52
19	Sleep disorders	1.12	−1.26	2.04	3.33
20	Feel like body had rotted away	1.70	1.11	2.23	3.30
21	Difficulties around	2.05	0.02	1.65	2.73
22	Future is promising	1.34	−1.11	0.52	2.18
23	Hypodynamia	1.65	−0.47	1.49	2.67
24	Difficult to start	1.48	−0.78	1.39	2.78
25	Sense of failure	2.62	0	1.31	2.12
26	Rapid heart beat	1.09	0.17	2.86	4.46
27	Immersion in the past	1.34	−1.14	1.06	2.48
28	Draw a blank	1.22	−1.20	1.13	2.98
29	Tiredness or fatigue	1.62	−1.30	1.78	3.09
30	Fear	1.99	−0.35	1.54	2.80
31	Indecisiveness	1.39	−1.02	1.10	2.70
32	Reasoning difficulty	1.64	−0.61	1.41	2.75
33	Mind blank	1.97	−0.17	1.54	2.60
34	Irresolution	1.44	−1.04	1.15	2.66
35	Clear mind	1.25	−1.33	0.60	2.37
36	Disappointment	2.64	−0.29	1.23	2.12
37	Poor appetite or eating too much	1.03	−1.54	2.41	3.96
38	Unattractiveness feelings	1.50	−0.83	1.33	2.63
39	Worse than others	1.99	−0.46	1.26	2.49
40	Self-assessment low	2.40	−0.10	1.38	2.38
41	Talk less	1.45	−0.63	1.33	2.68
42	Uncalm	2.27	−0.32	1.23	2.31
44	Not needed feelings	2.18	−0.21	1.29	2.26
45	Self-dislike	2.29	0.40	1.65	2.53
46	Loneliness	1.99	−0.48	1.12	2.03
47	Feel like crying	2.05	0.28	1.75	2.74
48	Self-criticalness	1.66	−0.75	2.13	2.96
49	Helplessness	2.25	−0.16	1.39	2.22
50	Unfriendly treatment feelings	1.55	0.19	2.34	3.60
51	Irritability	1.50	−0.31	1.73	3.18
52	Future is not appealing	2.56	0.41	1.60	2.44
53	Have no future	2.59	0.27	1.55	2.45
54	Suicidal thoughts	1.87	0.68	1.89	2.73
55	Concentration or memory difficulty	1.41	−1.01	2.21	3.49
56	Sadness	2.40	−0.64	1.15	2.03
57	Eating too much or little	0.99	0.09	2.66	4.29
58	Dilatory or intense behavior	1.31	−0.69	2.60	3.74
59	Restlessness	2.31	−0.25	1.33	2.31
60	Unpopularity	2.10	0.12	1.73	2.79
61	Others' life will be better without me	1.96	1.01	2.14	3.01
62	Smile less	1.87	−0.13	1.21	2.22
63	No good things	2.94	0.26	1.58	2.23
64	Despair	3.14	0.41	1.48	2.45
65	Unable to continue daily work	2.61	0.41	1.61	2.32
66	Regret and upset	1.71	−1.16	1.19	2.41
67	Unable to provide for oneself	1.81	0.38	2.07	3.08
68	Unable to restart	2.27	0.42	1.65	2.39

*a, Discrimination parameter; b, Threshold parameter*.

### CAT-depression simulation study

#### Characteristics of CAT-depression

Table 5 presents the CAT-Depression outcomes with the real individuals' dataset under different stopping rules. About 27.61 items on average (*SD* = 16.17) were administered for latent theta estimation under the stopping rule SE (theta) ≤ 0.2, while setting the stopping rule up to SE (theta)> 0.2 would lead to considerable further item savings (M_SE(theta) ≤ 0.3_ = 11.46, *SD*_SE (theta) ≤ 0.3_ = 9.57; M_SE(theta) ≤ 0.4_ = 6.48, *SD*_SE(theta) ≤ 0.4_ = 5.77; M_SE(theta) ≤ 0.5_ = 4.36, *SD*_SE(theta) ≤ 0.5_ = 3.53; (Table 5), and only a mean of 3.27 (*SD* = 1.58) was needed for latent theta estimation under the stopping rule SE (theta) ≤ 0.6. The Pearson's correlation between the estimated theta in the CAT-Depression and the estimated theta via the entire item bank ranged from 0.88 to 0.99 crossing a different stopping rule, which indicated that the adaptive algorithm was efficient for CAT-Depression.

**Table 5:** Characteristics of the CAT-Depression under several stopping rules.

**Stopping rule**	**Number of items used**		**Mean SE(theta)**	**Marginal reliability**	***r***
	**Mean**	**SD**			
None	68	0	0.15	0.97	1
SE (theta) ≤ 0.6	3.27	1.58	0.52	0.73	0.88
SE (theta) ≤ 0.5	4.36	3.53	0.45	0.79	0.90
SE (theta) ≤ 0.4	6.48	5.77	0.38	0.86	0.93
SE (theta) ≤ 0.3	11.46	9.57	0.29	0.91	0.96
SE (theta) ≤ 0.2	27.61	16.17	0.20	0.96	0.99

*None, the entire item bank was administered; r, the Pearson's correlation between the estimated theta in the CAT-Depression and the estimated theta via the entire item bank*.

**Figure 1:**
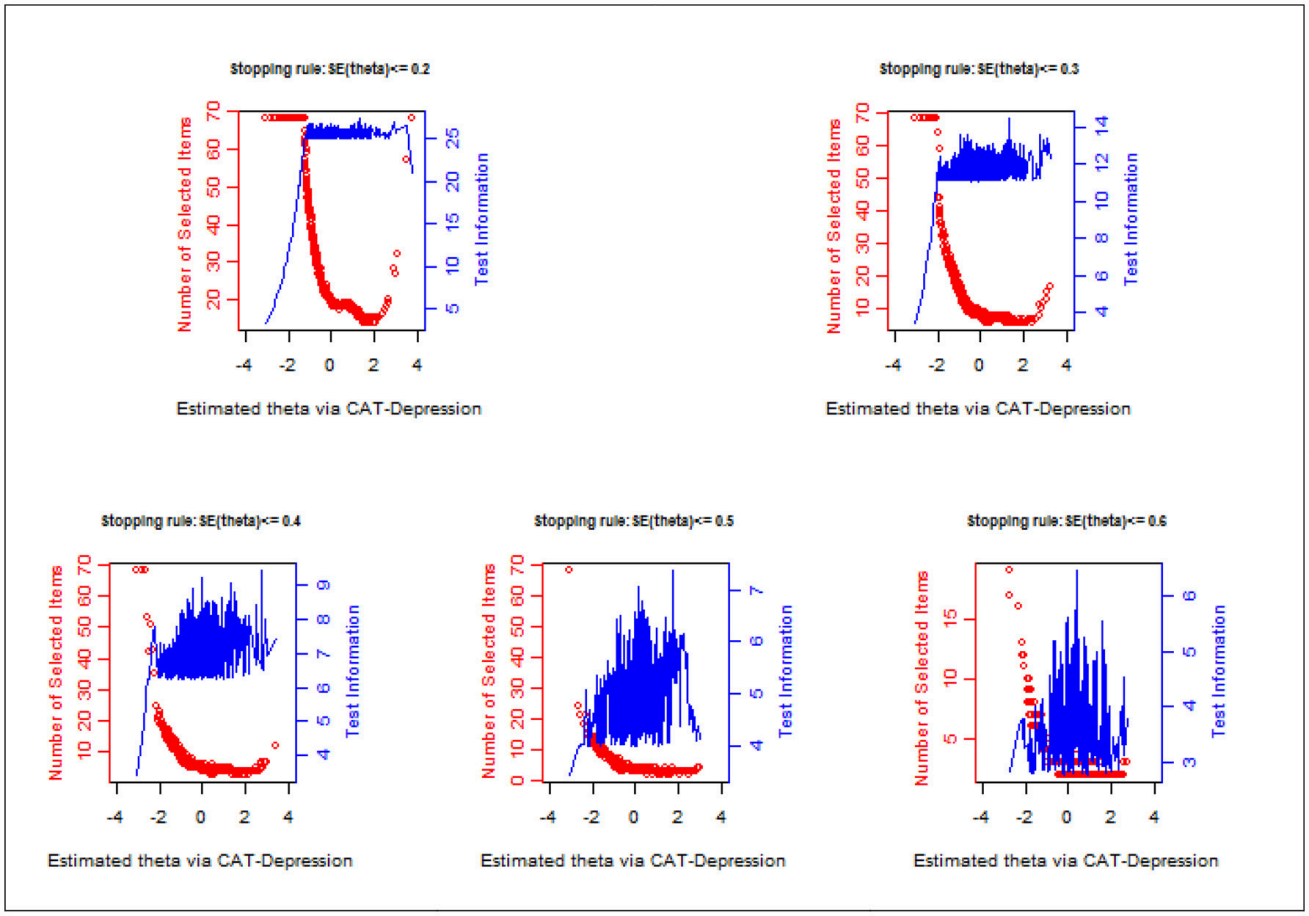
Number of selected items and test information curve under different stopping rules.

Figure 1 displays the number of administered items along with test information under each stopping rule. Evidently, a large number of items were administered for individuals with lower theta and the test information was lower. Fewer items were administered for individuals with middle or high theta and the test information was high. For example, under the stopping rule SE (theta) ≤ 0.2, (1) the test information was less than 10 for those whose theta ranged from −3 to −1.5 even if the entire item bank was administered to them; while (2) the test information was over 25 for those whose theta ranged from 0 to 2.5 with about 20 administered items to them.

**Figure 2:**
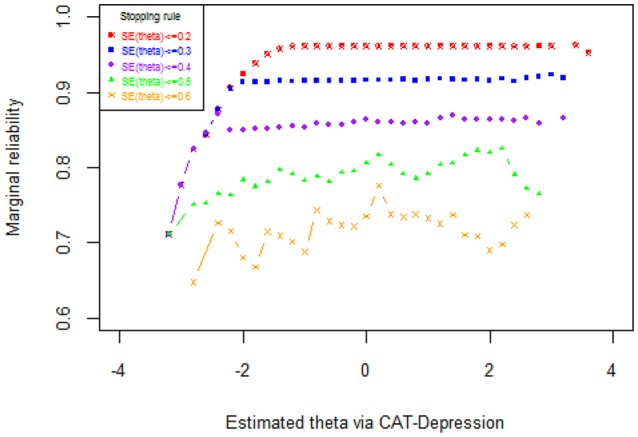
Reliability as a function of the theta under several stopping rules.

The results of the marginal reliability of CAT-Depression are documented in Figure 2 and Table 5. As shown in Table 5, the marginal reliabilities under different stopping rules varied from 0.73 to 0.97, with an average of 0.87, which were generally acceptable for individuals. Figure 2 displays the reliability for each individual under each stopping rule. Under two stopping rules, that is, SE (theta) ≤ 0.2 and SE (theta) ≤ 0.3, the reliabilities were above 0.9 for most individuals, and under the stopping rule SE (theta) ≤ 0.4 the reliabilities were above 0.85 (with an average of 0.96) for most individuals. These results again indicated that CAT-Depression had a high reliability for most individuals. In addition, the marginal reliability for individuals with theta estimation more than −2 under stopping rule SE (theta) ≤ 0.2 was maximal, while the marginal reliability under stopping rule SE (theta) ≤ 0.2, stopping rule SE (theta) ≤ 0.3, and stopping rule SE (theta) ≤ 0.4 were almost equal when theta estimation was less than −2. Individuals always had the minimum marginal reliability under stopping rule SE (theta) ≤ 0.6, regardless of theta estimation.

#### The content validity of CAT-depression

In the final item bank (*N* = 68), 13 items measured behavior-related depressive symptoms, 31 items measured cognition-related depressive symptoms, 16 items measured mood-related depressive symptoms, 5 items measured somatic-related depressive symptoms, and 3 items measured the symptom of suicidal thoughts according to the evaluation of the items in the item bank by five psychiatrists engaged in the diagnosis and treatment of depression for more than 10 years. All the symptoms in the *ICD-10* were measured, therefore the final item bank had good content validity.

#### The criterion-related validity of CAT-depression

The Pearson's correlations between CAT-Depression estimated theta and three depression-related scales (i.e., CES-D, SDS, and PHQ-9) were calculated to explore the criterion-related validity of CAT-Depression. As documented in Table 6, the Pearson's correlations between CAT-Depression and CES-D ranged from 0.82 to 0.94 under different stopping rules while the Pearson's correlations with SDS and PHQ-9 ranged from 0.74 to 0.83 and from 0.66 to 0.74, respectively. These results indicated that CAT-Depression had an acceptable and reasonable criterion-related validity no matter which type of stopping rule was used.

**Table 6:** Criterion-related validity of CAT-Depression with external criteria scales under different stopping rules.

**Stopping rule**	**CES-D (95% CI)**	**SDS (95% CI)**	**PHQ-9 (95% CI)**
None	0.941(0.934–0.947)	0.836(0.817–0.852)	0.740(0.713–0.766)
SE (theta) ≤ 0.6	0.825(0.806–0.843)	0.742(0.715–0.767)	0.641(0.605–0.674)
SE (theta) ≤ 0.5	0.838(0.820–0.855)	0.752(0.725–0.776)	0.663(0.629–0.695)
SE (theta) ≤ 0.4	0.867(0.851–0.880)	0.771(0.746–0.794)	0.684(0.651–0.714)
SE (theta) ≤ 0.3	0.895(0.883–0.906)	0.793(0.770–0.814)	0.689(0.657–0.718)
SE (theta) ≤ 0.2	0.922(0.913–0.930)	0.814(0.793–0.833)	0.713(0.683–0.740)

*95% CI, 95% confidence intervals; None, the entire item bank was administered*.

#### The predictive utility (sensitivity and specificity) of CAT-depression

The ROC analysis for CAT-Depression is presented in Table 7. Results of the CAT's diagnostic accuracy based on the CES-D, SDS, and PHQ-9 scales revealed that the AUC values based on the three scales were 0.977 (sensitivity = 0.905, specificity = 0.930, cut-off = 0.025), 0.898 (sensitivity = 0.787 specificity = 0.866, cut-off = −0.465), and 0.886 (sensitivity = 0.764, specificity = 0.860, cut-off = −0.275), respectively, when no stopping rule was applied. Then the AUC values dropped to 0.916 (sensitivity = 0.855, specificity = 0.804, cut-off = −0.025), 0.884 (sensitivity = 0.707, specificity = 0.837, cut-off = −0.232), and 0.831 (sensitivity = 0.645, specificity = 0.873, cut-off = −0.024), respectively, conditional on a stopping rule of SE (theta) ≤ 0.6. Even so, the AUC values under all stopping rules were also higher than the critical value 0.7, which is universally used as the lower bound for moderate predictive utility. Overall, the sensitivity and specificity of CAT-Depression were both acceptable. For example, the sensitivity and specificity of CAT-Depression were 0.937 and 0.851, respectively, under the stopping rule of SE (theta) ≤ 0.2 while using the CES-D as the classification criteria for depression.

**Table 7:** The predictive utility (sensitivity and specificity) of the CAT-Depression under different stopping rules.

**Stopping rule**	**CES-D**					**SDS**					**PHQ-9**				
	**AUC (95%CI)**	**Cut-off**	**Se**	**Sp**	**YI**	**AUC(95%CI)**	**Cut-off**	**Se**	**Sp**	**YI**	**AUC(95%CI)**	**Cut-off**	**Se**	**Sp**	**YI**
None	0.977(0.971–0.984)	0.025	0.905	0.930	0.835	0.898(0.878–0.918)	−0.465	0.787	0.866	0.653	0.886(0.867–0.905)	−0.275	0.764	0.860	0.624
SE (theta) ≤ 0.6	0.916(0.901–0.932)	−0.025	0.855	0.804	0.659	0.844(0.817–0.871)	−0.232	0.707	0.837	0.544	0.831(0.806–0.856)	−0.024	0.645	0.873	0.518
SE (theta) ≤ 0.5	0.919(0.904–0.935)	−0.019	0.848	0.822	0.670	0.851(0.826–0.876)	−0.537	0.822	0.733	0.555	0.833(0.808–0.858)	−0.012	0.611	0.893	0.504
SE (theta) ≤ 0.4	0.938(0.925–0.951)	−0.105	0.926	0.805	0.731	0.856(0.832–0.881)	−0.314	0.720	0.856	0.577	0.847(0.824–0.871)	−0.020	0.651	0.890	0.54
SE (theta) ≤ 0.3	0.951(0.940–0.962)	−0.013	0.879	0.869	0.749	0.869(0.846–0.892)	−0.306	0.732	0.861	0.593	0.852(0.828–0.875)	−0.084	0.679	0.883	0.562
SE (theta) ≤ 0.2	0.963(0.954–0.972)	−0.094	0.937	0.851	0.788	0.886(0.864–0.907)	−0.455	0.788	0.851	0.639	0.865(0.843–0.886)	−0.125	0.682	0.886	0.568

*95% CI, 95% confidence intervals; None, No stopping rule was applied; AUC, Area Under Curve; Se, Sensitivity; Sp, Specificity; YI, Youden-Index*.

## Discussion

In this study, CAT-Depression was developed using the GRM in a Chinese sample and then the characteristics, marginal reliability, criterion-related validity, predictive utility (sensitivity and specificity), and diagnostic performance of CAT-Depression were investigated.

To construct a high-quality item bank for CAT-Depression, items were carefully selected from ten widely-used depression scales based on the symptom criteria of depression in *ICD-10*. Then, a strict unidimensionality local independence check was conducted to examine whether the assumptions of the IRT models were met. Moreover, five commonly used polytomous IRT models were compared based on real data to select one optimal model for CAT-Depression. Finally, analyses of item-fit, discrimination, and DIF were carried out to construct a high-quality item bank. Results showed that (1) the final unidimensional item bank included 68 items, and these items had local independence, good item fit, high discrimination, no DIF, and each item measured at least one symptom criterion of depression in *ICD-10*; (2) the mean IRT discrimination of the item bank reached 1.784, which clearly showed that the final item bank of CAT-Depression was high-quality; (3) the results of CAT-Depression revealed that about 11.46 items on average were used to estimate depression under stopping rule SE (theta) ≤ 0.3, while only about 4.36 items were needed with stopping rule SE (theta) ≤ 0.5, and fewer items were administered for individuals with middle or high theta. Additionally, the test information/reliability was high. Test information curve plots (Figure 1) revealed that the information of the depression item bank peaked on the right side of the depression theta scale, and a larger number of or even all selected items were needed for patients with a lower theta. Therefore, in the context of individuals with a similar degree of depression, small differences are more easily detected for respondents with high scores than for respondents with low scores of depression. This result is similar to previous studies (Smits et al., 2011). Reise and Waller (2009) believe psychopathology constructs may be unipolar, which would have led to this result. Moreover, we believe this phenomenon was normal, as the main goal of CAT-Depression is to diagnose the severity of depression rather than to diagnose the health of patients. Thus, it may provide more information for persons with depression, to diagnose them more precisely.

To further investigate the marginal reliability, criterion-related validity, and predictive utility (sensitivity and specificity) of CAT-Depression, a CAT simulation study with real data was carried out. The results revealed that: (1) CAT-Depression had an acceptable marginal reliability with an average of 0.87, ranging from 0.73 to 0.97; (2) CAT-Depression had reasonable and acceptable criterion-related validities (ranging from 0.66 to 0.94) with the CES-D, SDS, and PHQ-9. The criterion-related validity and diagnostic performance were greatest when the CES-D was used as the criterion scale. This may have been caused by most of the theta values of the patients in this study, which ranged from −2 to 2 (Only 4.4% of the patients were outside this range). Umegaki and Todo (2017) study showed that the CES-D scale provided more information than that of the SDS and the PHQ-9 within the range of about −2 to 2 of depression severity; (3) the sensitivity and specificity of the CAT-Depression were both acceptable, and especially the sensitivity and specificity of the CAT-Depression were 0.937 and 0.851, respectively, under the stopping rule SE (theta) ≤ 0.2 when using the CES-D as the classification criteria for depression. In addition, ROC curves analysis indicated that the diagnostic performance of the CAT theta affected by setting the stopping rule up was negligible [AUC_(CES−D)_ = 0.916–0.977; AUC_(SDS)_ = 0.844–0.898; AUC_(PHQ−9)_ = 0.831–0.886]. CAT had good screening performance, and the AUC value was higher (0.831–0.977) than the value of the lower bound for a moderate predictive utility under all stopping rules. The sensitivity (0.611–0.937) and specificity (0.733–0.930) of CAT-Depression were both acceptable. The minimum probability that a patient was accurately diagnosed with a disease, and that general people were accurately diagnosed with no illness were 0.611 and 0.733, respectively, which were higher than the random level (0.5).

Although this study revealed that CAT-Depression had acceptable reliability, validity (including reasonable sensitivity and specificity), and good diagnostic performance, there were some limitations. The distribution of item content was generally uneven, 31 items measured cognition-related depressive symptoms and 16 items measured mood-related depressive symptoms, but only 5 items measured somatic-related depressive symptoms and 3 items measured suicidal ideation in the final depression item bank. The accuracy and validity of assessment for individuals with cognition-related and mood-related depressive symptoms are slightly higher than for individuals with somatic-related depressive symptoms and suicidal thoughts. However, depression had an important predictive effect on the morbidity and mortality of somatic diseases (Bush et al., 2001; Di et al., 2006) and suicidal ideation indicated a very severe depressive state. Future studies should add some items to CAT-Depression to address these issues. Additionally, a CAT simulation study with real response data was conducted in this article; however, a real CAT administration should be conducted in future research to deeply explore the efficiency of CAT-Depression. Different results may be produced by simulated and real CAT administration (Smits et al., 2011) in that there are many factors that affect individuals' responses in a real situation, such as answering time, individual mood, test environment, etc. Fortunately, a prior study showed that the results of simulated CAT were in line with actual CAT (Kocalevent et al., 2009). As a consequence, the present article still has some practical significance. Finally, the simulation study in this study indicated that the two stopping rules, SE (theta) ≤ 0.3 and SE (theta) ≤ 0.4, may be the most economical, precise, and valid stopping rules, and may be the best for actual CAT on depression administration. Future studies may investigate the economy, precision, and validity of CAT-Depression in practice.

## Author contributions

QT thesis writing. YC and DT guide the thesis writing and data processing. QL and YZ data processing.

### Conflict of interest statement

The authors declare that the research was conducted in the absence of any commercial or financial relationships that could be construed as a potential conflict of interest.
